# Phylogeny and Evolutionary History of Respiratory Complex I Proteins in Melainabacteria

**DOI:** 10.3390/genes12060929

**Published:** 2021-06-18

**Authors:** Christen Grettenberger, Dawn Y. Sumner, Jonathan A. Eisen, Anne D. Jungblut, Tyler J. Mackey

**Affiliations:** 1 Department of Earth and Planetary Sciences, University of California Davis, Davis, CA 95616, USA; dysumner@ucdavis.edu; 2 Genome Center, University of California Davis, Davis, CA 95616, USA; jaeisen@ucdavis.edu; 3 Life Sciences Department, The Natural History Museum, London Sw7 5BD, UK; ajungblut@nhm.ac.uk; 4 Department of Earth and Planetary Sciences, University of New Mexico, Albuquerque, NM 87131, USA; tjmackey@umn.edu

**Keywords:** aerobic respiration, phylogenomics, Melainabacteria, Sericytochromatia, Cyanobacteria, evolution

## Abstract

The evolution of oxygenic photosynthesis was one of the most transformative evolutionary events in Earth’s history, leading eventually to the oxygenation of Earth’s atmosphere and, consequently, the evolution of aerobic respiration. Previous work has shown that the terminal electron acceptors (complex IV) of aerobic respiration likely evolved after the evolution of oxygenic photosynthesis. However, complex I of the respiratory complex chain can be involved in anaerobic processes and, therefore, may have pre-dated the evolution of oxygenic photosynthesis. If so, aerobic respiration may have built upon respiratory chains that pre-date the rise of oxygen in Earth’s atmosphere. The Melainabacteria provide a unique opportunity to examine this hypothesis because they contain genes for aerobic respiration but likely diverged from the Cyanobacteria before the evolution of oxygenic photosynthesis. Here, we examine the phylogenies of translated complex I sequences from 44 recently published Melainabacteria metagenome assembled genomes and genomes from other Melainabacteria, Cyanobacteria, and other bacterial groups to examine the evolutionary history of complex I. We find that complex I appears to have been present in the common ancestor of Melainabacteria and Cyanobacteria, supporting the idea that aerobic respiration built upon respiratory chains that pre-date the evolution of oxygenic photosynthesis and the rise of oxygen.

## 1. Introduction

Early in Earth’s history, in the Archaean, organisms evolved the ability to perform oxygenic photosynthesis [1,2,3,4,5,6]. The byproduct of this metabolic innovation, O_2_, led to a second transformative innovation, aerobic respiration, the use of previously trace amounts of O_2_ to fuel metabolic activities. This highly effective metabolism was co-opted by much of life and paved the way for multicellular species [7]. 

Aerobic respiration proceeds through four complexes, the last two of which (complexes III and IV) interact directly with O_2_. As they interact with O_2_, complexes III and IV likely emerged after the advent of oxygenic photosynthesis. Phylogenetic studies of these complexes from across the bacterial tree of life support the hypothesis that they evolved after the great oxidation event (GOE) [8,9]. However, there is some disagreement. For example, an analysis of 673 bacterial and archaeal genomes showed that different terminal oxidases (complex IV subunits) had different evolutionary trajectories and, therefore, likely originated at different times. One terminal oxidase (A-O_2_Red) may have been present before the divergence of major bacterial and archaeal phyla and thus before the evolution of oxygenic photosynthesis [10].

Unlike complexes III and IV, complex I does not interact with O_2_. Therefore, its evolutionary history is less likely to be linked to the rise of oxygen in Earth’s atmosphere. Complex I can be involved in anaerobic processes, including denitrification. The inclusion of complex I in both denitrification and aerobic respiration suggests that these two respiratory chains may share an evolutionary history [11,12,13]. One hypothesis posits that the earliest respiratory chain was composed of complex I, a quinone pool, and a terminal electron acceptor and that this respiratory chain eventually gave rise to both denitrification and aerobic respiration [11]. Phylogenetics can lend insight into the evolutionary history of complex I, the timing of its origination, and whether it may have been a part of this hypothesized respiratory chain. However, to the best of our knowledge, there have been no phylogenetic studies of the genes in complex I. 

The discovery of the Melainabacteria significantly aided efforts to use phylogenetics to understand the evolution of oxygenic photosynthesis and aerobic respiration [8,9]. The Melainabacteria is a non-photosynthetic sister group to the other Cyanobacteria. The naming and the classification of these groups are currently under debate (see discussion in [14]). For ease, here we will refer to the phototrophic Cyanobacteria as “Cyanobacteria,” members of the phylum or class Melainabacteria or Vampirovibrionia as “Melainabacteria,” and members of the phylum or class Sericytochromatia as “Sericytochromatia”. These groups share a suite of genes that they inherited from their common ancestor. Genes present in one group but not the other (e.g., those involved in photosynthesis) result from either origination in one lineage or loss from the others. Multiple members of the Melainabacteria contain the genes necessary for aerobic respiration, and those genes can provide insight into whether the evolution of complex I predated the divergence of the Melainabacteria and Cyanobacteria, and thus the GOE. Overall, genes that were present in the common ancestor of the Cyanobacteria and Melainabacteria and vertically transmitted in both lineages will have phylogenies that match the "species" phylogenies constructed using translated single-copy genes. Those genes acquired via lateral gene transfer after the separation of the Cyanobacteria and Melainabacteria would likely have phylogenies that do not match the translated single-copy gene phylogeny. Previous work has examined the phylogenies of complex III and IV proteins of the Melainabacteria, Cyanobacteria, and other bacteria [8,9]. Phylogenetically, the melainabacterial genes for these two complexes are most closely related to non-cyanobacterial taxa, suggesting that the Melainabacteria acquired them via lateral gene transfer after the divergence of the Melainabacteria and Cyanobacteria [8,9]. Complex I phylogenies should mirror the translated single-copy gene-based phylogeny if they pre-date the divergence of the Melainabacteria and Cyanobacteria and were inherited via lineal descent. Otherwise, the phylogenies should suggest lateral gene transfer, as is seen in complexes III and IV.

Here, we use recently released metagenome assembled genomes (MAGs) from the Genomes from Earth’s Microbiome Catalog (GEMs). We use the phylogenies of translated gene sequences involved in aerobic respiration from these MAGs, other Melainabacteria, the Cyanobacteria, and other bacterial groups to examine the evolutionary history of the genes involved in aerobic respiration within the melainabacterial and cyanobacterial lineages. 

## 2. Materials and Methods 

We retrieved 198 genomes, including members of the Melainabacteria and Sericytochromatia, and genomes that contained respiratory complexes from across the bacterial tree of life. We retrieved Melainabacteria and Sericytochromatia genomes by selecting all genomes available in the Genomes from Earth’s Microbiome Catalog (GEMs) [15]. All other publicly available Melainabacteria and Sericytochromatia genomes that contained genes for aerobic respiration were also used. Non-melainabacterial, non-Sericytochromatia genomes were chosen by selecting one genome from each of the bacterial orders available on Integrated Microbial Genomes and Microbiomes (IMG) [16]. We also selected additional genomes from early branching Cyanobacteria including the Gloeobacterales and *Gloeomargarita.* These taxa were selected because of their phylogenetic position. Genomes that were selected and had nucleotide data available for download were used for the rest of the study. Genome quality was assessed using CheckM v.1.0.7 [17]. These genomes were annotated using PROKKA 1.13 [18] and we retrieved the translated nucleotide sequences for sequences annotated as complex I genes *nuoA*, *nuoB*, *nuoC*, *nuoD*, *nuoH*, *nouI*, *nuoJ*, *nuoL*, *nuoM*, and *nuoN*. *nuoK* was not well annotated by PROKKA so we retrieved a Hidden Markov Model (HMM) for *nuoK* from eggNOG 5.0.0 [19]. We used this model to retrieve the corresponding translated nucleotide sequences in Anvi’o 6.2 using an E-value of 1 × 10^−30^ [20]. Both of these methods often retrieved related but non-target sequences. Therefore, we annotated the retrieved sequences using GhostKOALA v. 2.2 and selected only those annotated as the protein of interest [21]. A list of the genomes used that contained complex I genes, their accession numbers, and the number of copies of complex I genes is available in Supplemental Table S1.

Sequences were aligned using MAFFT v. 7.471 on the CIPRES Science Gateway. Regions where the alignment was >50% gaps were trimmed using TrimAI v. 1.2.59 on XSEDE [22]. The best fit model of protein substitution was identified using ModelTest-NG v. 0.1.5 on XSEDE on the CIPRES Science Gateway. The candidate model was set to have discrete Gamma rate categories (+G). We did not allow for a proportion of invariant sites (+I) because this is not recommended for RAxML [23]. We constructed a maximum likelihood tree using RAxML-HPC2 on XSEDE (v 8.2.12) on the CIPRES Science Gateway using 1000 bootstrap iterations using the best fit model selected using BIC within ModelTest-NG [23] (Supplemental Table S2). Trees were visualized in Interactive Tree of Life (iTOL) v. 6 [24]. The trees were not rooted because we do not know the evolutionary history of, and thus an appropriate root for, these proteins. 

All cyanobacterial genomes contained multiple copies of *nuoL* and *nuoM*, consistent with the previous literature [25,26]. Therefore, we excluded them from the concatenated complex I tree described below. Fifty of the genomes contained multiple copies of other complex I genes. We cannot create a concatenated gene tree that includes genes that are present in multiple copies with different evolutionary histories. Therefore, we selected the genomes that contained only a single copy of *nuoA*, *nuoB*, *nuoC*, *nuoD*, *nuoH*, *nuoI*, *nuoJ*, *nuoK*, and *nuoN* and contained at least 7 of these 9 genes, 102 genomes in total (Supplemental Table S1). We created a concatenated gene tree of translated *nuoA*, *nuoB*, *nuoC*, *nuoD*, *nuoH*, *nuoI*, *nuoJ*, *nuoK*, and *nuoN* for these genomes. A maximum likelihood tree was constructed in RAxML-HPC2 on XSEDE (v 8.2.12) on the CIPRES Science Gateway as described above. Each gene used the amino acid substitution model identified using ModelTest-NG. Trimmed alignments, tree files, and iTOL annotation files are available on the Open Science Framework (OSF) [27]. 

Single-copy, marker-genes from the Anvi’o bacterial_71 gene set were retrieved using Anvi’o 6.2 following the Anvi’o tutorial on phylogenomics [28]. Genes were aligned using MUSCLE v. 3.8.1551 within Anvi’o 6.2 and concatenated [29]. We constructed a maximum likelihood tree using RAxML-HPC2 on XSEDE (v 8.2.12) on the CIPRES Science Gateway using 1000 bootstrap iterations and standard parameters including a Protein CAT model, DAYHOFF protein substitution matrix, and no correction for ascertainment bias [30,31]. The tree was visualized in iTOL [24] and is unrooted. 

## 3. Results

**Figure 1:**
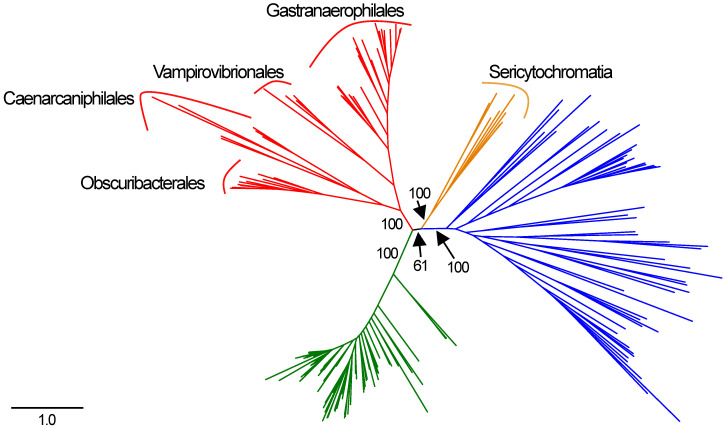
Concatenated, single-copy marker gene tree constructed using the Bacteria_71 collection of single-copy core genes from Anvi’o [20]. Cyanobacteria are indicated in green, Melainabacteria in red, Sericytochromatia in orange, and other phyla in blue. Order-level divisions are indicated for the Melainabacteria. Bootstrap values are indicated for key splits.

**Figure 2:**
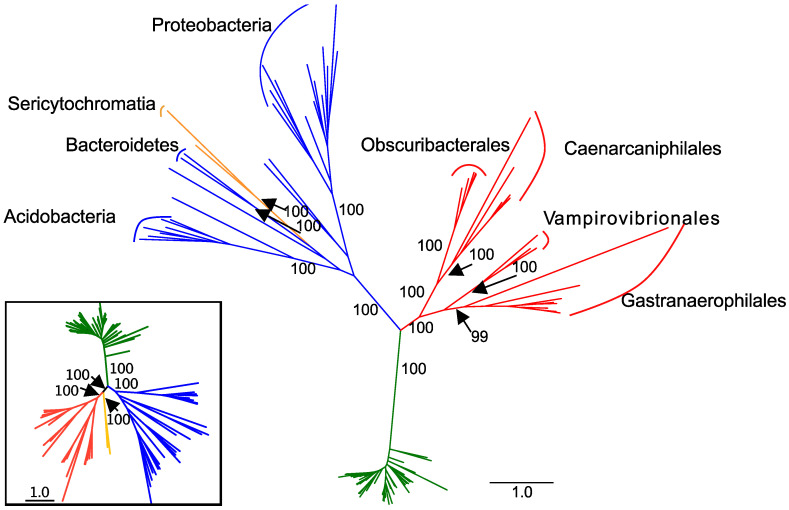
Maximum likelihood tree constructed from the concatenation of translated *nuoA*, *nuoB*, *nuoC*, *nuoD*, *nuoH*, *nuoI*, *nuoJ*, *nuoK*, and *nuoN* sequences (3358 amino acid residues). The tree contains 102 taxa. Cyanobacteria are indicated in green, Melainabacteria in red, Sericytochromatia in orange, and other groups in blue. For non-Cyanobacteria, phyla with 2 or more representatives are labeled. Order-level classifications are indicated within the Melainabacteria. Bootstrap values are indicated for labeled clades. Inset: concatenated, single-copy phylogeny containing only the 102 genomes used to construct the concatenated tree in the main figure.

We retrieved 198 genomes that contained genes encoding complex I proteins. Genomes were retrieved from from 15 phyla (Supplemental Table S1; Figure 1). From the GEMs catalog, we retrieved five Sericytochromatia and 44 Melainabacteria MAGs. Of the Melainabacteria MAGs from the GEMs catalog, six are from the Caenarcaniphilales, 22 are from the Gastranaerophilales, 11 from the Obscuribacterales, and five from the Vampirovibrionales. Of these MAGs, 14 did not contain any genes for complex I proteins. A total of 102 genomes from seven phyla contained no duplicated target genes and were used in the final concatenated complex I gene tree. The concatenated tree contained eight Actinobacteria, two Bacteroidetes, one Deferribacteres, one Nitrospirae, 13 Proteobacteria, one Verrucomicrobia, 48 Cyanobacteria, two Sericytochromatia and 26 Melainabacteria—six Caenarcaniphilales, 11 Gastranaerophilales, five Obscuribacteriales, and four Vampirovibrionales (Figure 2). The remaining MAGs either contained fewer than seven genes annotated as encoding complex I proteins or contained multiple copies of at least one complex I gene (excluding *nuoL* or *nuoM*). 

### Phylogeny of Complex I

In the concatenated complex I tree, the Melainabacteria form a monophyletic clade that branches between the Cyanobacteria and the non-cyanobacterial, non-melainabacterial taxa. This division has strong bootstrap support (Figure 2). This branching pattern is congruent with their phylogenetic placement as a sister group to the Cyanobacteria (Figure 1). Within the Melainabacteria, there are four monophyletic clades, each of which correlate with order-level divisions (Figure 2). The two Sericytochromatia MAGs used in the concatenated complex I tree are most closely related to non-cyanobacterial species. This is not congruent with their phylogenetic placement in the concatenated, single-copy gene tree where they appear as a sister group to the Melainabacteria (Figure 1 and Figure 2). 

The single gene complex I trees, excluding the translated *nuoK*, *nuoL* and *nuoM* trees, are similar to one another and generally mirror the topology of the concatenated complex I tree (Supplemental Figures S1–S8). In these trees, the Cyanobacteria generally form a monophyletic clade with 88% or higher bootstrap support. For example, the cyanobacterium *Hassallia byssoidea* has multiple copies of all complex I genes except for *nuoI* and *nuoK*. When multiple gene copies are present, one or more copies of each gene are most closely related to a cyanobacterial species and one or more are most closely related to a non-cyanobacterial species. In the translated *nuoB* tree, *Mastigocladus laminosus* UU774 contains a single gene copy but is most closely related to non-cyanobacterial taxa. In these trees, the Melainabacteria form either a single large clade (translated *nuoB*, *nuoD*, *nuoH*, *nuoI*, *nuoJ*, and *nuoN* sequences) or up to four smaller clades (translated *nuoA* and *nuoC* sequences). The Sericytochromatia are most closely related to non-cyanobacterial taxa. The division between Melainabacteria and other taxa has lower bootstrap support in individual trees than in the concatenated trees. 

In the translated *nuoA*, *nuoB*, *nuoH*, *nuoI*, *nuoJ*, and *nuoN* trees, one or more non-cyanobacterial taxa are most closely related to either the Melainabacteria or the Cyanobacteria and are not congruent with their phylogenetic placement in the single-copy concatenated gene tree. More than 75% of the time, these taxa contain multiple copies of the gene of interest and at least one copy of the gene of interest is most closely related to non-cyanobacterial taxa. 

In the translated *nuoK* tree, the Melainabacteria are not monophyletic. Instead, one clade emerges in the same phylogenetic position as in the concatenated tree and an additional clade is most closely related to cyanobacterial species (Supplemental Figure S9). A single Melainabacteria MAG is most closely related to non-cyanobacterial, non-melainabacterial species. 

The translated *nuoL* and *nuoM* trees share a similar phylogenetic structure that is different from the other translated *nuo* trees (Supplemental Figures S10 and S11). Most Cyanobacteria (>95%) contain at least two copies of *nuoL* and *nuoM*. The sequences from the cyanobacterial genomes form two separate clades in phylogenies of translated *nuoL* and *nuoM* sequences. In the translated *nuoM* tree, the Melainabacteria form a monophyletic group and the non-cyanobacterial phyla form a separate clade. In both phylogenies, the Sericytochromatia sequences are most closely related to non-melainabacterial, non-cyanobacterial taxa. The translated *nuoL* phylogeny mirrors that of the translated *nuoM* phylogeny, except it contains an additional clade made up primarily (>85%) of taxa that contain multiple copies of *nuoL.* Unlike the Cyanobacteria, less than 15% of melainabacterial species and approximately one third of taxa from other phyla contain multiple copies of *nuoL* or *nuoM* (Supplemental Figures S10 and S11; Supplemental Table S1).

## 4. Discussion

### Possible Evolutionary History of Complex I

We can gain insight into the origin and evolution of aerobic respiration and the structure of the early aerobic respiratory chain by looking at differences in the evolutionary histories of complex I, which is involved in anaerobic processes, compared to complexes III and IV, which are only engaged in aerobic ones [11,12,13]. The Melainabacteria are a critical group in this comparison because they likely diverged from the Cyanobacteria 2.5–3.1 GA, before the evolution of oxygenic photosynthesis and the rise of oxygen [1,2]. If the earliest respiratory chains were simple and composed of complex I, a quinone pool, and a “simple” complex IV as previously predicted [11], we would expect that the phylogeny of complex I would mirror that of single-copy genes. 

The complex I phylogenies largely support the hypothesis that complex I proteins were vertically, rather than laterally, transmitted in the Melainabacteria and Cyanobacteria. The melainabacterial complex I proteins are monophyletic with strong bootstrap support for their position and for order-level divisions within the group (Figure 2). Therefore, their phylogeny mirrors that of the concatenated single-copy gene tree (Figure 1 and Figure 2). This pattern supports the hypothesis that complex I predated the division of the Cyanobacteria and the Melainabacteria and the divisions of the orders within the Melainabacteria. However, in some individual translated nucleotide trees, one or more non-cyanobacterial taxa branch between the Cyanobacteria and the Melainabacteria. Most of these sequences (>75%) are from a taxon that (1) contains multiple copies of that gene, and (2) at least one copy of the gene is phylogenetically cohesive with other members of its phylum. Both the *H. byssoidea* and *M. laminosus* genomes appear to contain contamination [32] (Table S1), indicating that some of these sequences may be due to contamination within the genome. Alternately, if the sequences do belong to the species, those that are not phylogenetically placed with other members of their phyla could be the result of lateral gene transfer. 

However, phylogenies of translated *nuoL* and *nuoM* have a different topology from other translated complex I genes. Translated *nuoL* and *nuoM* contain two distinct clades of Cyanobacteria (Supplemental Figures S10 and S11). Many Cyanobacteria have two copies of these genes (>95%), which is consistent with previous work [25], and each genome often contains one sequence in each clade. This pattern may indicate a gene duplication or lateral gene transfer event early in the evolutionary history of these genes, or it may be caused by misannotation. Previous phylogenetic studies of cyanobacterial *nuoL* and *nuoM* genes indicate that the two genes may be related via a gene duplication event that eventually led to two different types of NuoL and NuoM found in Cyanobacteria [33]. Additionally, *Synechocystis* sp. PCC 6803 use different versions of NuoL and NuoM under ambient and low CO_2_ conditions, indicating that these types may have different physiological roles [33]. However, not all cyanobacterial genomes contain multiple copies of these genes, and these genes are closely related to a large cation/proton antiporter family and are occasionally misannotated [25,26]. Therefore, in some cases, the presence of duplicate sequences may be due to misannotation rather than the presence of multiple copies in the genome. Disentangling these two potential explanations will likely require examining the expression in each species. 

The Sericytochromatia are a sister group to the Cyanobacteria/Melainabacteria clade in both the full and reduced concatenated single-copy phylogenies (Figure 1 and Figure 2). Therefore, their phylogeny should mirror that of the translated single-copy gene phylogeny if the complex I genes originated before the divergence of the Sericytochromatia and the Cyanobacteria/Melainabacteria clade. However, it does not (Figure 2). Unlike the Melainabacteria, the location of the Sericytochromatia translated complex I genes differs from the clade’s position in the translated single-copy gene phylogeny. The concatenated complex I gene tree contains approximately half the number of taxa as the original dataset, and the number of Sericytochromatia genomes is reduced from eight to two. The reduction in the number of species may influence the phylogeny. However, in the single-copy gene tree constructed with only the 102 genomes used in the concatenated complex I tree, the Sericytochromatia appear as a sister group to the Melainabacteria, mirroring the phylogeny of the full dataset (Figure 1 and Figure 2). Additionally, in phylogenies of individual complex I proteins, the Sericytochromatia are not most closely related to the Melainabacteria. Therefore, we hypothesize that the phylogenetic pattern seen in the concatenated complex I tree is reflective of the evolutionary history of complex I within this group rather than an artifact of the reduced tree size. This likely indicates that the Sericytochromatia received the genes for these proteins via lateral gene transfer. This pattern may have resulted in one of two ways: (1) complex I emerged after the divergence of the Cyanobacteria/Melainabacteria group from the Sericytochromatia and the Sericytochromatia received complex I via later gene transfer at a later time, or (2) complex I emerged before this divergence but was lost in the common ancestor of all known Sericytochromatia and was then later regained via lateral gene transfer.

## 5. Conclusions

Our study provides new data supporting the origination of complex I before the divergence of the Cyanobacteria and Melainabacteria and possibly after the divergence of the Sericytochromatia from the melainabacterial/cyanobacterial clade. This supports the hypothesis that the earliest respiratory chain originated before aerobic respiration and contained complex I [11]. After oxygen became locally available and complexes III and IV evolved, they were likely laterally transferred into the Melainabacteria [8,9]. Species could perform aerobic respiration by building upon the existing respiratory chain [32] in a “Lego-like” fashion—using each complex as a building block that could be built upon by or paired with other complexes [34]. In the case of aerobic respiration, this modular structure may have allowed organisms not capable of aerobic respiration to gain that ability by receiving the genes for appropriate complex III and IV proteins rather than requiring an entirely new respiratory chain.

## Data Availability

Alignments and tree files can be accessed on the Open Science Framework at https://osf.io/b72ym/?view_only=ca528ff7c74e42babfefa82ab25b9b50 (accessed on 18 June 2021).

## Supplementary Materials

The following are available online: https://www.mdpi.com/article/10.3390/genes12060929/s1, Table S1: Genomes used for phylogenetic analyses, Table S2: Genes retrieved for phylogenetic analysis, the number of leaves in the resulting phylogenetic tree, the number of amino acid residues in the trimmed alignment, and the model used for each maximum likelihood tree, Supplemental Figure S1: Maximum likelihood tree of translated *nuoA* sequences. The tree contains 190 leaves and is based on 120 amino acid residues. Cyanobacteria are indicated in green, Melainabacteria in red, Sericytochromatia in orange, and other taxa in blue. Organisms with multiple copies of *nuoA* are indicated by grey squares, Supplemental Figure S2: Maximum likelihood tree of translated *nuoB* sequences. The tree contains 193 leaves and is based on 236 amino acid residues. Cyanobacteria are indicated in green, Melainabacteria in red, Sericytochromatia in orange, and other taxa in blue. Organisms with multiple copies of *nuoB* are indicated by grey squares, Supplemental Figure S3: Maximum likelihood tree of translated *nuoC* sequences. The tree contains 155 leaves and is based on 171 amino acid residues. Cyanobacteria are indicated in green, Melainabacteria in red, Sericytochromatia in orange, and other taxa in blue. Organisms with multiple copies of *nuoC* are indicated by grey squares, Supplemental Figure S4: Maximum likelihood tree of translated *nuoD* sequences. The tree contains 153 leaves and is based on 393 amino acid residues. Cyanobacteria are indicated in green, Melainabacteria in red, Sericytochromatia in orange, and other taxa in blue. Organisms with multiple copies of *nuoD* are indicated by grey squares, Supplemental Figure S5: Maximum likelihood tree of translated *nuoH* sequences. The tree contains 196 leaves and is based on 362 amino acid residues. Cyanobacteria are indicated in green, Melainabacteria in red, Sericytochromatia in orange, and other taxa in blue. Organisms with multiple copies of *nuoH* are indicated by grey squares, Supplemental Figure S6: Maximum likelihood tree of translated *nuoI* sequences. The tree contains 177 leaves and is based on 194 amino acid residues. Cyanobacteria are indicated in green, Melainabacteria in red, Sericytochromatia in orange, and other taxa in blue. Organisms with multiple copies of *nuoI* are indicated by grey squares, Supplemental Figure S7: Maximum likelihood tree of translated *nuoJ* sequences. The tree contains 139 leaves and is based on 203 amino acid residues. Cyanobacteria are indicated in green, Melainabacteria in red, Sericytochromatia in orange, and other taxa in blue. Organisms with multiple copies of *nuoJ* are indicated by grey squares, Supplemental Figure S8: Maximum likelihood tree of translated *nuoJ* sequences. The tree contains 202 leaves and is based on 493 amino acid residues. Cyanobacteria are indicated in green, Melainabacteria in red, Sericytochromatia in orange, and other taxa in blue. Organisms with multiple copies of *nuoJ* are indicated by grey squares, Supplemental Figure S9: Maximum likelihood tree of translated *nuoK* sequences. The tree contains 157 leaves and is based on 102 amino acid residues. Cyanobacteria are indicated in green, Melainabacteria in red, Sericytochromatia in orange, and other taxa in blue, Supplemental Figure S10: Maximum likelihood tree of translated *nuoL* sequences. The tree contains 348 leaves and is based on 582 amino acid residues. Cyanobacteria are indicated in green, Melainabacteria in red, Sericytochromatia in orange, and other taxa in blue. Organisms with multiple copies of *nuoL* are indicated by grey squares, Supplemental Figure S11: Maximum likelihood tree of translated *nuoM* sequences. The tree contains 459 leaves and is based on 502 amino acid residues. Cyanobacteria are indicated in green, Melainabacteria in red, Sericytochromatia in orange, and other taxa in blue. Organisms with multiple copies of *nuoM* are indicated by grey squares.
