# Supplementary material for: Phylogeny and Evolutionary History of Respiratory Complex I Proteins in Melainabacteria
Source: Genes (Basel). 2021 Jun 18;12(6):929. doi: 10.3390/genes12060929 (PMC8235220; doi:10.3390/genes12060929)
Supplement: Supplementary file 1 [file genes-12-00929-s001.zip › GenesFinalSupplemental/Supplemental Figure 1.pdf]

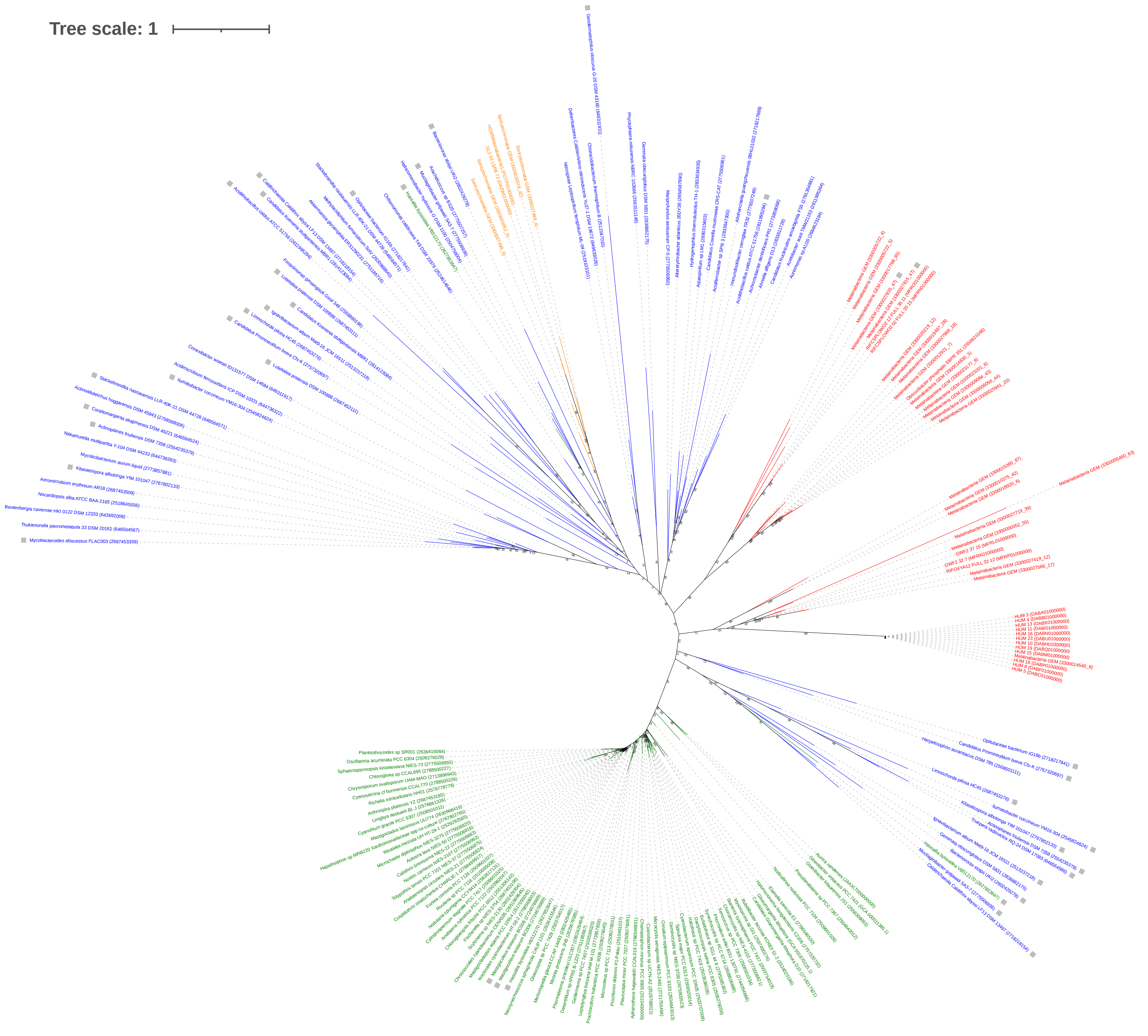
Tree scale: 1
Mycobacteroides abscessus FLAC003 (2687453359)
Tsukamurella paurometabola 33 DSM 20162 (646564587)
Beutenbergia cavernae HKI 0122 DSM 12333 (643692008)
Nocardiopsis alba ATCC BAA-2165 (2518645556)
Aeromicrobium erythreum AR18 (2687453509)
HUM 3 (DABA01000000)
HUM 4 (DABB01000000)
HUM 13 (DABK01000000)
HUM 11 (DABI01000000)
HUM 16 (DABN01000000)
HUM 23 (DABU01000000)
HUM 10 (DABH01000000)
HUM 19 (DABQ01000000)
HUM 15 (DABM01000000)
HUM 8 (DABF01000000)
HUM 5 (DABC01000000)
