# Supplementary figures and images for: Phylogeny and Evolutionary History of Respiratory Complex I Proteins in Melainabacteria
Source: Genes (Basel). 2021 Jun 18;12(6):929. doi: 10.3390/genes12060929 (PMC8235220; doi:10.3390/genes12060929)

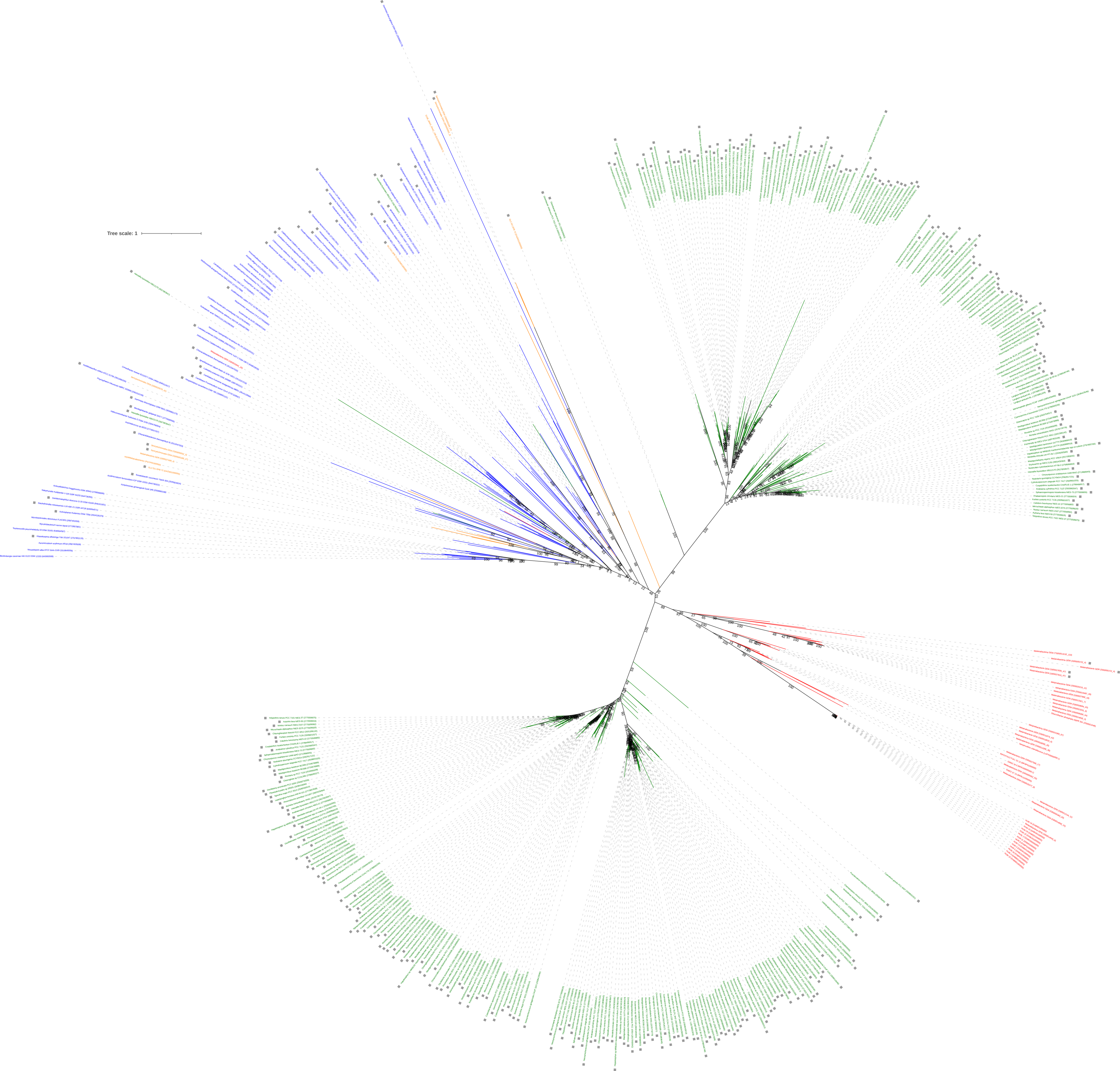

Supplement: Supplementary file 1 [file genes-12-00929-s001.zip › GenesFinalSupplemental/Supplemental Figure 11.pdf]

Tree scale: 1

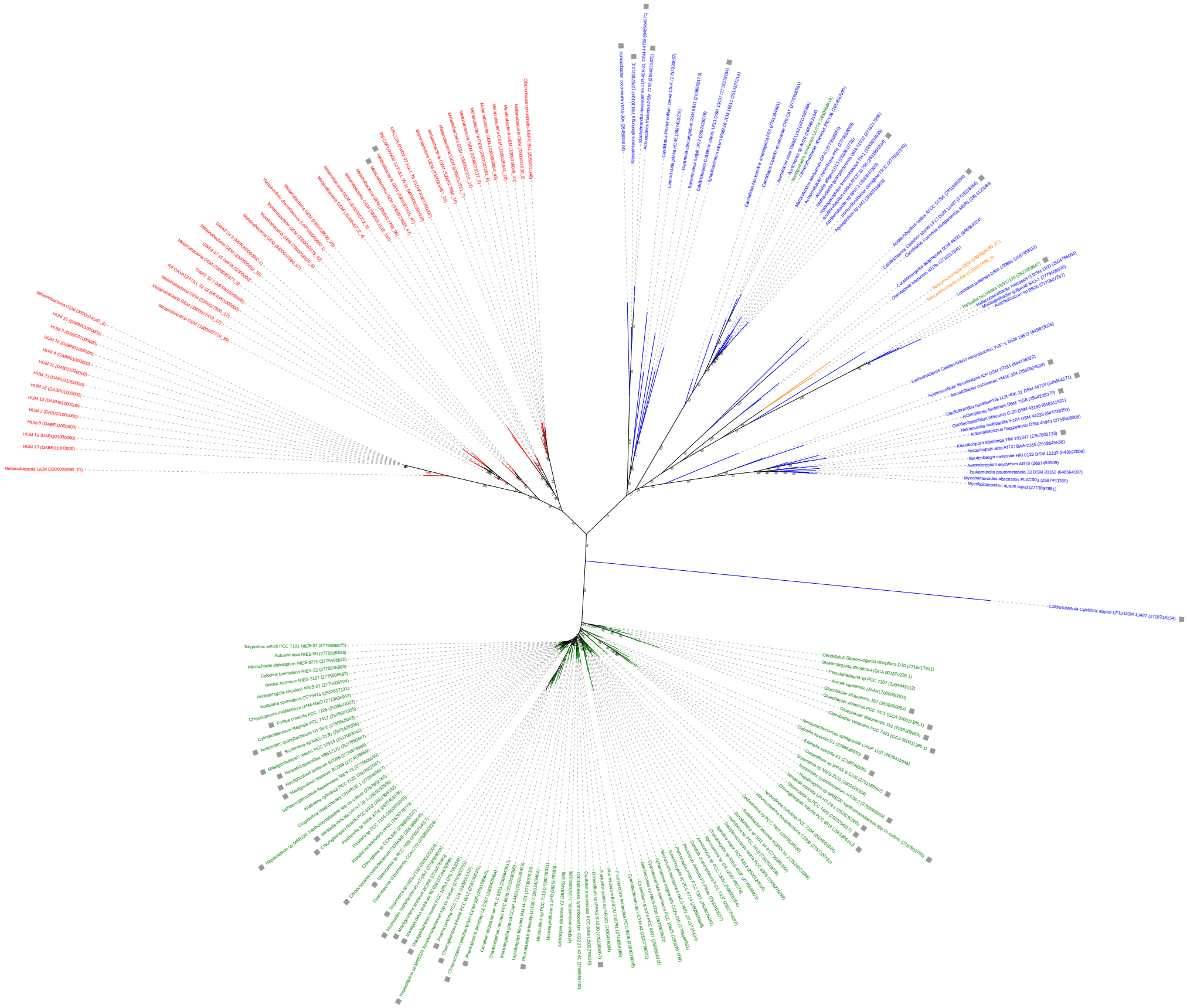

Supplement: Supplementary file 1 [file genes-12-00929-s001.zip › GenesFinalSupplemental/Supplemental Figure 2.pdf]

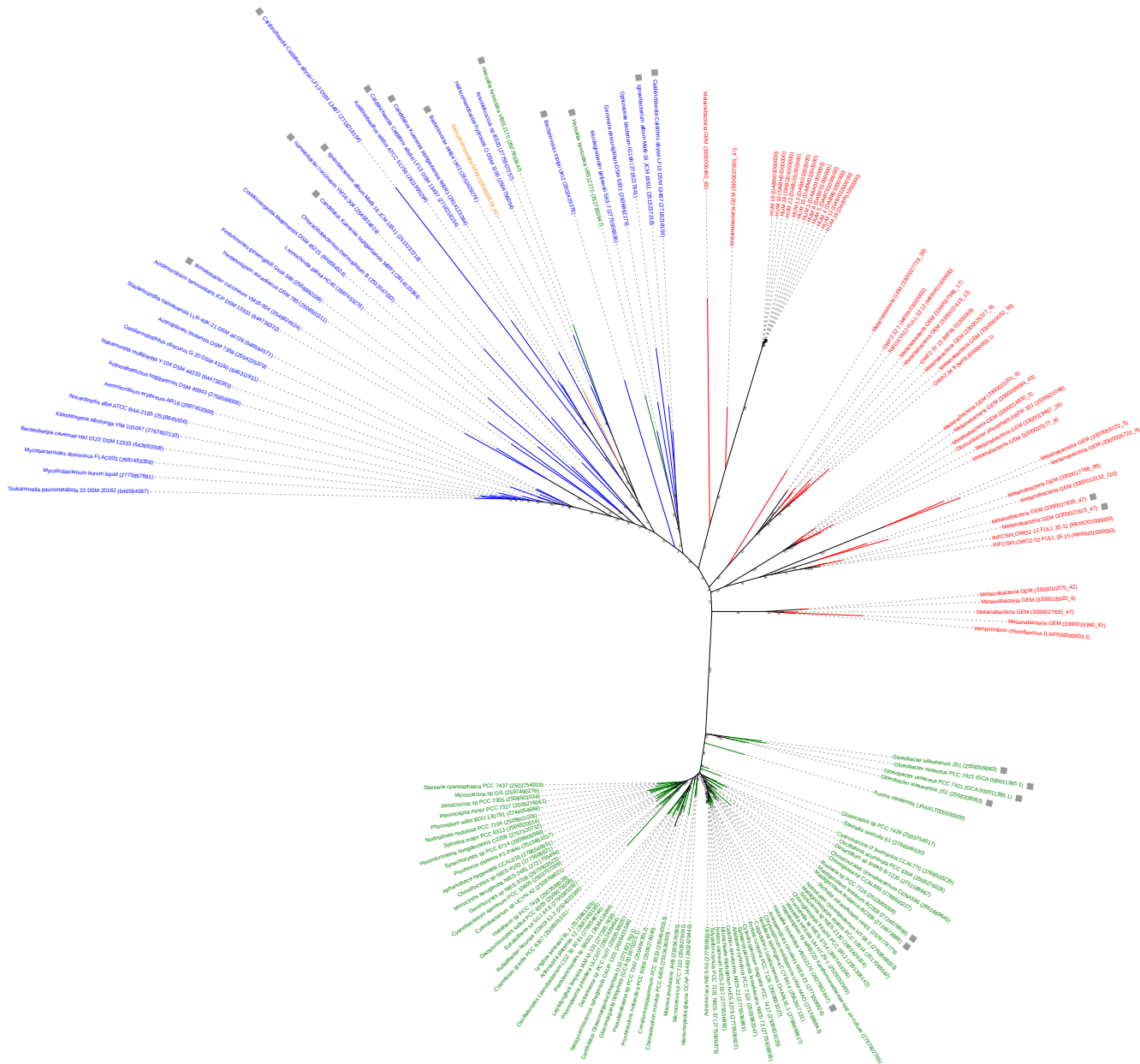

Supplement: Supplementary file 1 [file genes-12-00929-s001.zip › GenesFinalSupplemental/Supplemental Figure 3.pdf]

Tree scale: 1 

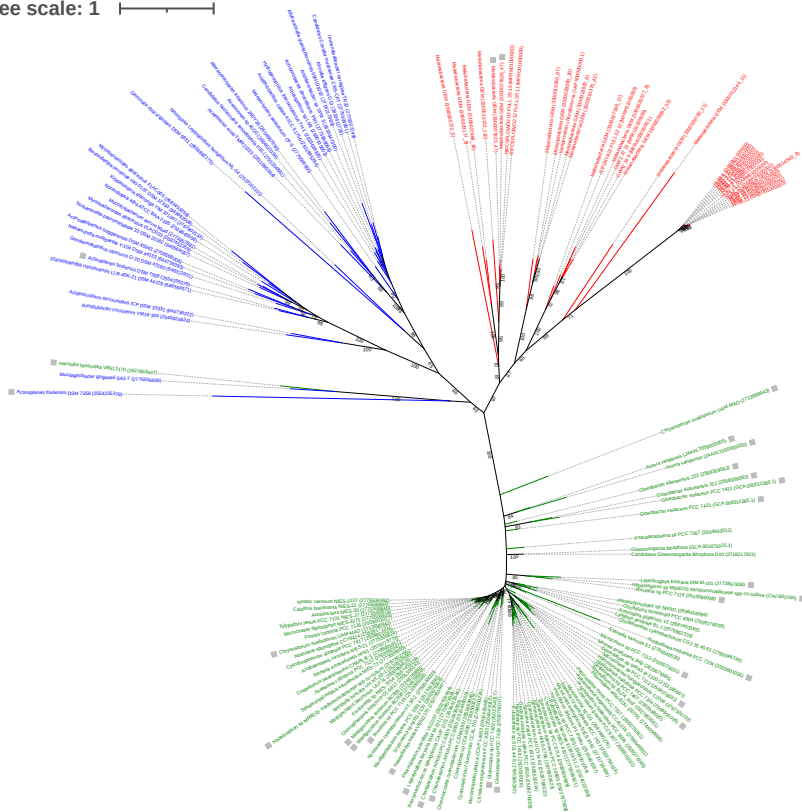

Supplement: Supplementary file 1 [file genes-12-00929-s001.zip › GenesFinalSupplemental/Supplemental Figure 4.pdf]

Tree scale: 1 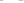

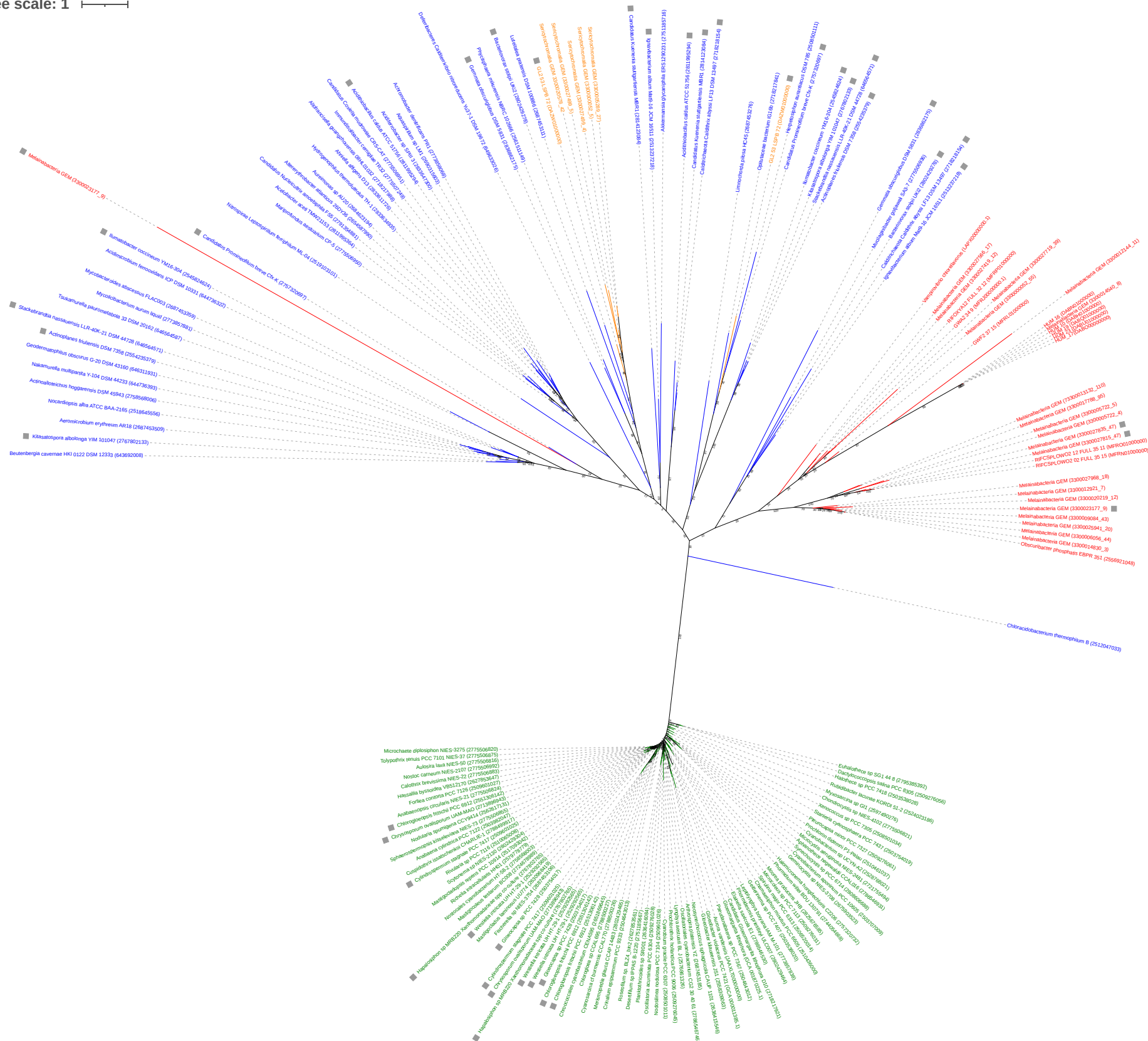

Supplement: Supplementary file 1 [file genes-12-00929-s001.zip › GenesFinalSupplemental/Supplemental Figure 6.pdf]

Tree scale: 1 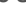

Supplement: Supplementary file 1 [file genes-12-00929-s001.zip › GenesFinalSupplemental/Supplemental Figure 7.pdf]

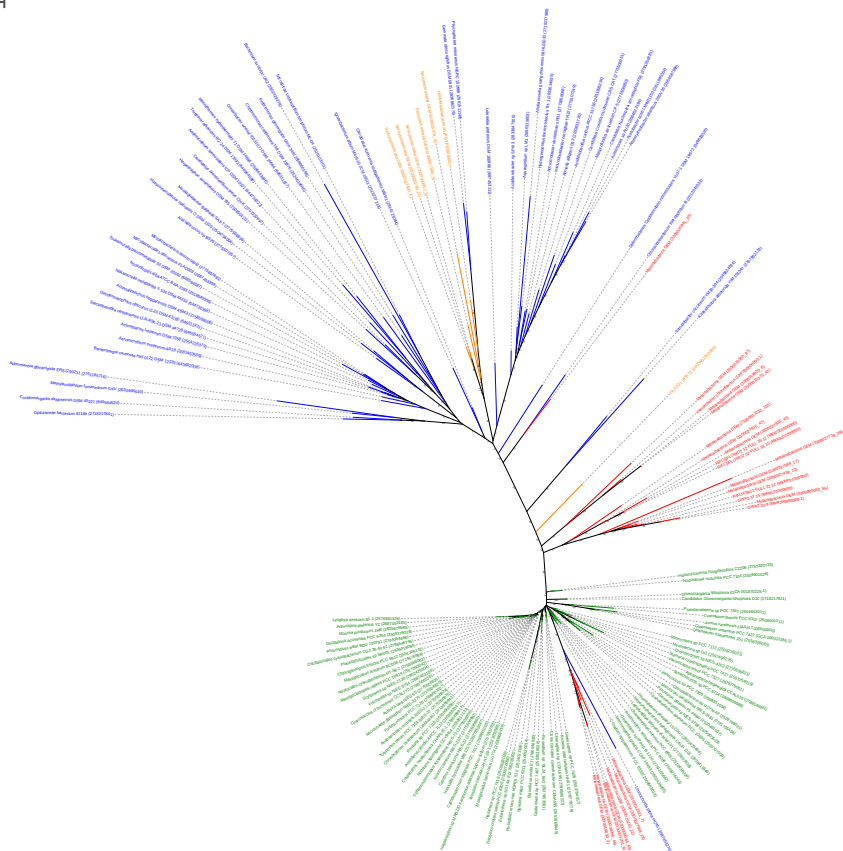

Supplement: Supplementary file 1 [file genes-12-00929-s001.zip › GenesFinalSupplemental/Supplemental Figure 9.pdf]
