# Supplementary material for: Phylogeny and Evolutionary History of Respiratory Complex I Proteins in Melainabacteria
Source: Genes (Basel). 2021 Jun 18;12(6):929. doi: 10.3390/genes12060929 (PMC8235220; doi:10.3390/genes12060929)
Supplement: Supplementary file 1 [file Supplemental Figure 5.pdf › GenesFinalSupplemental/Supplemental Figure 5.pdf]

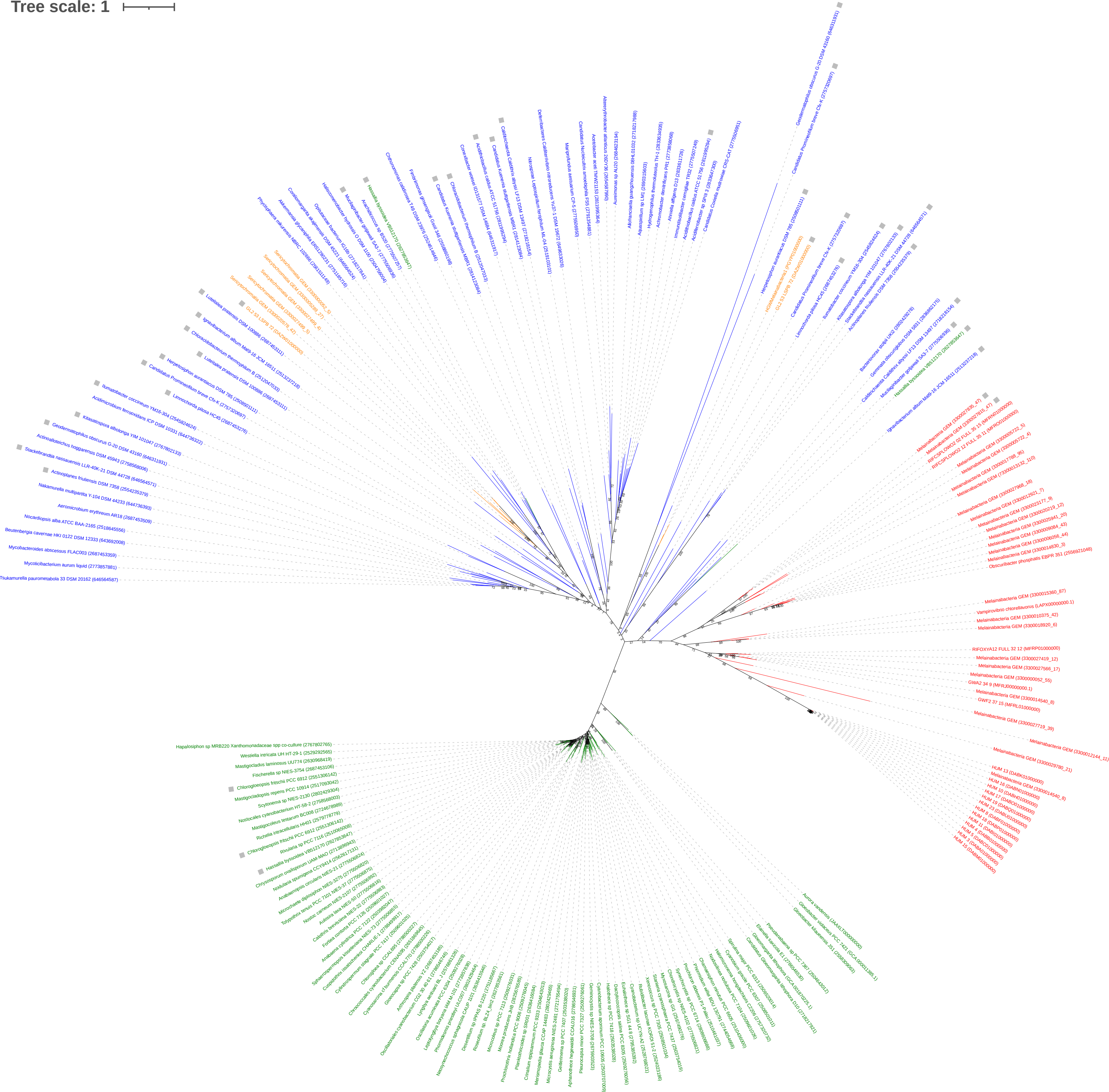

Tree scale: 1
Tsukamurella paurometabola 33 DSM 20162 (646564587)
Mycolicibacterium aurum liquid (2773857881)
Mycobacteroides abscessus FLAC003 (2687453359)
Beutenbergia cavernae HKI 0122 DSM 12333 (643692008)
Nocardiopsis alba ATCC BAA-2165 (2518645556)
Aeromicrobium erythreum AR18 (2687453509)
Nakamurella multipartita Y-104 DSM 44233 (644736393)
Actinoplanes friuliensis DSM 7358 (2554235379)
Stackebrandtia nassauensis LLR-40K-21 DSM 44728 (646564571)
Melainabacteria GEM (3300015360_87)
Vampirovibrio chlorellavorus (LAPX00000000.1)
Melainabacteria GEM (3300010375_42)
Melainabacteria GEM (3300018920_6)
RIFOXYA12 FULL 32 12 (MFRP01000000)
Melainabacteria GEM (3300027419_12)
Melainabacteria GEM (3300027566_17)
Melainabacteria GEM (3300000052_55)
GWA2 34 9 (MFRJ00000000.1)
Melainabacteria GEM (3300014540_8)
GWF2 37 15 (MFRL01000000)
Melainabacteria GEM (3300027719_39)
Obscuribacter phosphatis EBPR 351 (2556921048)
Hapalosiphon sp MRB220 Xanthomonadaceae spp co-culture (2767802765)
Westiella intricata UH HT-29-1 (2529292565)
Mastigocladus laminosus UU774 (2630968419)
Fischerella sp NIES-3754 (2687453106)
