# Supplementary material for: Phylogeny and Evolutionary History of Respiratory Complex I Proteins in Melainabacteria
Source: Genes (Basel). 2021 Jun 18;12(6):929. doi: 10.3390/genes12060929 (PMC8235220; doi:10.3390/genes12060929)
Supplement: Supplementary file 1 [file Supplemental Figure 8.pdf › GenesFinalSupplemental/Supplemental Figure 8.pdf]

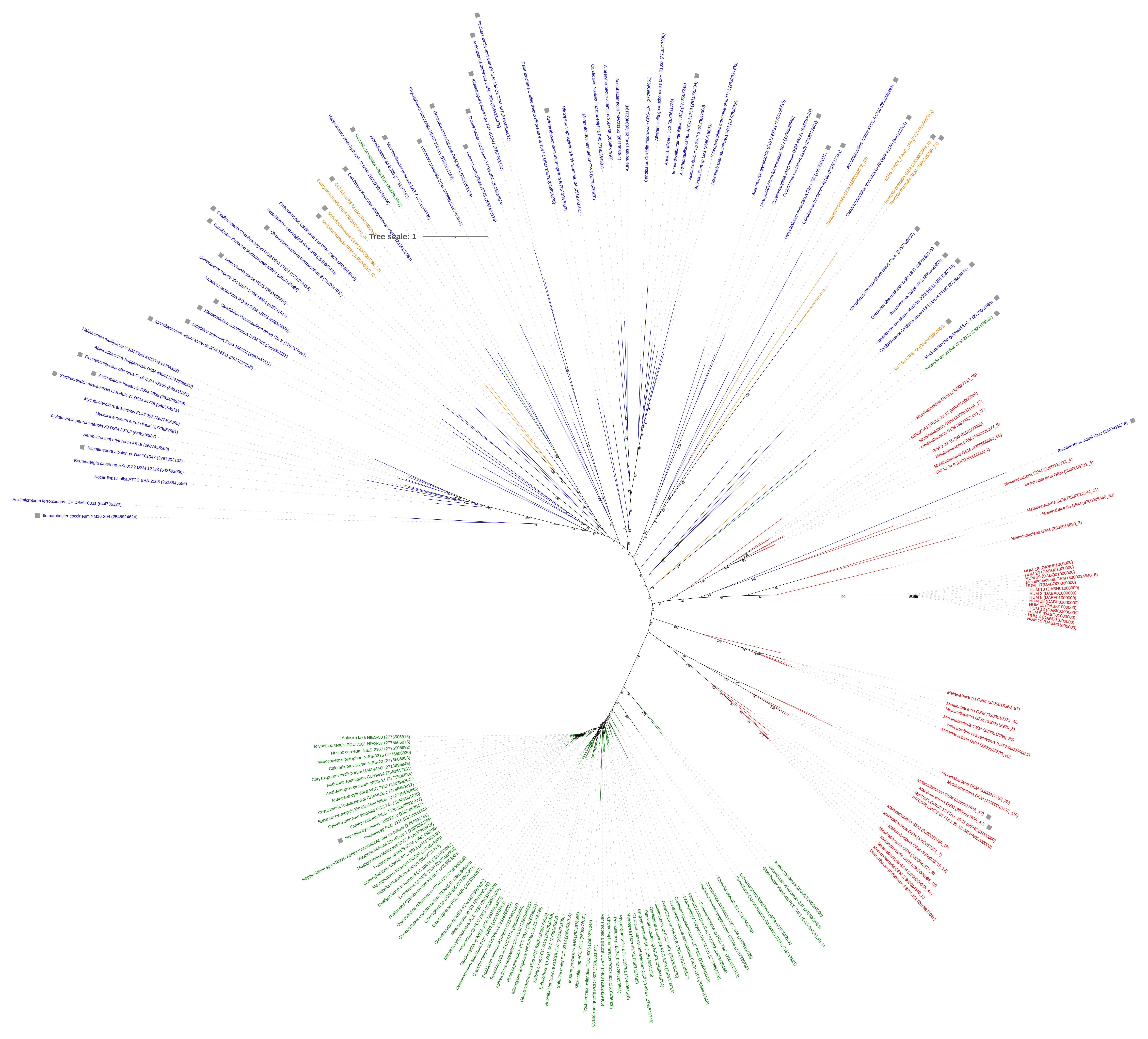

Tree scale: 1
Acidimicrobium ferrooxidans ICP DSM 10331 (644736322)
Ilumatobacter coccineum YM16-304 (2545824624)
Nocardiopsis alba ATCC BAA-2165 (2518645556)
Beutenbergia cavernae HKI 0122 DSM 12333 (643692008)
Kitasatospora albolonga YIM 101047 (2767802133)
Aeromicrobium erythreum AR18 (2687453509)
Tsukamurella paurometabola 33 DSM 20162 (646564587)
Mycolicibacterium aurum liquid (2773857881)
Mycobacteroides abscessus FLAC003 (2687453359)
Bacteriovorax stolpii Uki2 (2802429278)
Melainabacteria GEM (3300005722_4)
Melainabacteria GEM (3300005722_5)
Melainabacteria GEM (3300012144_11)
Melainabacteria GEM (3300005460_63)
Melainabacteria GEM (3300014830_3)
HUM 16 (DABN01000000)
HUM 23 (DABU01000000)
HUM 19 (DABQ01000000)
Melainabacteria GEM (3300014540_8)
HUM 17(DABO00000000)
HUM 10 (DABH01000000)
HUM 3 (DABA01000000)
HUM 8 (DABF01000000)
HUM 18 (DABP01000000)
HUM 11 (DABI01000000)
HUM 13 (DABK01000000)
HUM 5 (DABC01000000)
HUM 4 (DABB01000000)
HUM 15 (DABM01000000)
